# Abdominal Wall Hematoma Secondary to Dissection of the Deep Circumflex Iliac Artery: A Rare Complication of Ultrasound-Guided Paracentesis

**DOI:** 10.7759/cureus.5935

**Published:** 2019-10-17

**Authors:** Shivantha Amarnath, Magda Daoud, Stephen M Mulrooney

**Affiliations:** 1 Internal Medicine, Staten Island University Hospital, Northwell Health, Staten Island, USA; 2 Gastroenterology, Staten Island University Hospital, Northwell Health, Staten Island, USA

**Keywords:** deep circumflex iliac artery, abdominal wall hematoma, paracentesis, inferior epigastric artery, angiographic embolization

## Abstract

Paracentesis of the abdominal cavity is carried out to analyze ascitic fluid for diagnostic and therapeutic purposes. In recent years, the modern ultrasound-guided method is favored over the landmark-based approach as the latter carries a higher risk of complications. Dissection of the inferior epigastric artery is the most frequent complication encountered on either approach. We present a unique case of iatrogenic laceration of the deep circumflex iliac artery due to abnormal vessel anatomy in a patient with ascites.

## Introduction

Aspiration of ascitic fluid via paracentesis is performed for both diagnostic and therapeutic purposes, either using a landmark-based approach (LBA) or the safer ultrasound-guided method. The complication rate for paracentesis is less than 2%, and the LBA carries a nearly four-fold increased risk as opposed to the sonographic approach. Complications can include ascites leak, bowel perforation, infection/peritonitis, and bleeding. It is well documented that hemorrhagic complications due to accidental transection of the inferior epigastric artery (IEA) or its branches can lead to abdominal wall hematomas, pseudoaneurysms, and hemoperitoneum. Herein, we report a rare complicated case of accidental iatrogenic laceration of the deep circumflex iliac artery (DCIA) due to aberrant vessel anatomy in a patient with ascites.

## Case presentation

A 50-year-old female with a history of decompensated alcoholic liver cirrhosis with coagulopathy, thrombocytopenia, and ascites (Child-Pugh class C) presented with progressively worsening abdominal distention and discomfort for one week. She also endorsed a weight gain of 10 pounds and generalized weakness. The patient had undergone screening upper endoscopy and colonoscopy six months prior to admission. Endoscopy did not reveal any evidence of peptic ulcer disease, esophageal or gastric varices. Colonoscopy only demonstrated three diminutive polyps that were resected. She denied any fever, vomiting, diarrhea, red blood per rectum, confusion, or sleep dysregulation.

Vitals were within normal limits, and physical exam was significant for a soft distended abdomen with intact bowel sounds and fluid shift without any rigidity or guarding. Mental status was intact without any overt focal neurological deficits or asterixis. Laboratory studies were notable for thrombocytopenia of 118 K/uL, international normalized ratio (INR) of 2.5 (patient was not on any anticoagulants), alkaline phosphatase of 167 U/L, alanine aminotransferase of 98 U/L, aspartate aminotransferase of 167 U/L, albumin of 2.2 g/dL, and total bilirubin of 15.4 mg/dL with indirect bilirubin of 9 mg/dL.

**Figure 1:**
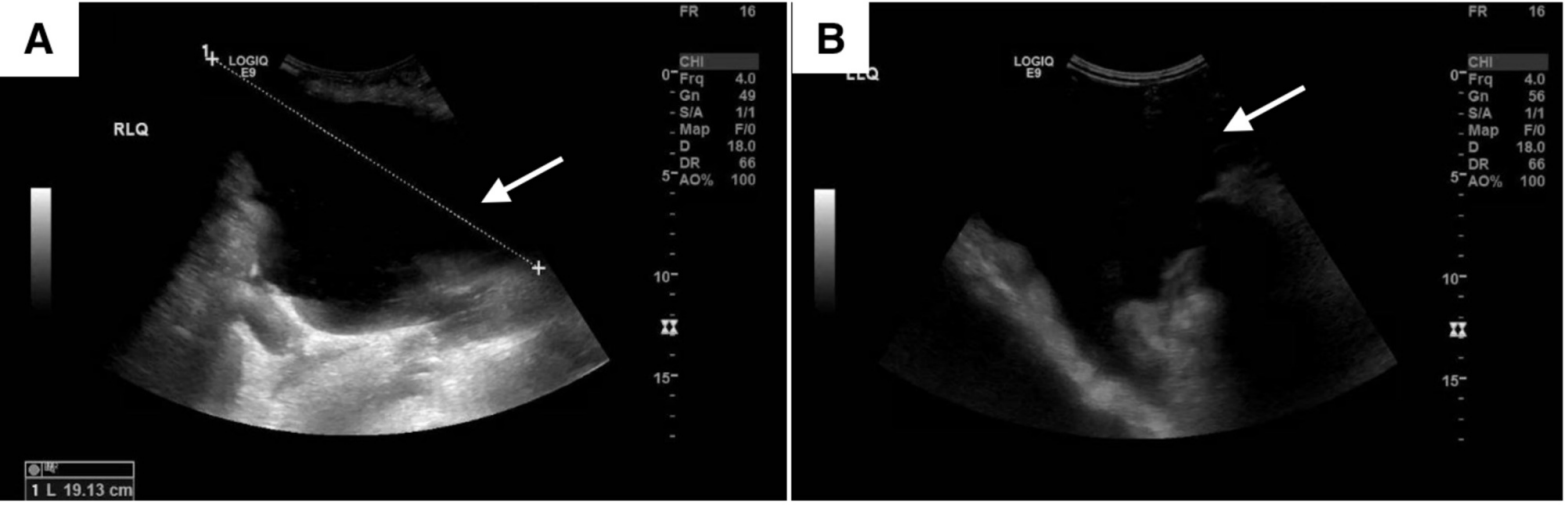
Sonographic evidence of large abdominopelvic ascites of the right lower quadrant (A) and left lower quadrant (B). The largest pocket is 19.13 cm in the right lower quadrant.

She was admitted for worsening ascites due to decompensated liver cirrhosis with a model for end-stage liver disease (MELD) score of 29. The patient was started on a 2g per day sodium-restricted diet along with furosemide 40mg and spironolactone 100mg daily. Ultrasound imaging of the abdomen demonstrated extensive abdominal and pelvic ascites with the largest pocket in the right lower quadrant measuring up to 19 cm in maximal dimension (Figure 1). She underwent a bedside ultrasound-guided diagnostic paracentesis of the right lower quadrant using a 20-gauge needle with drainage of 60 ccs of serous fluid. 

**Figure 2:**
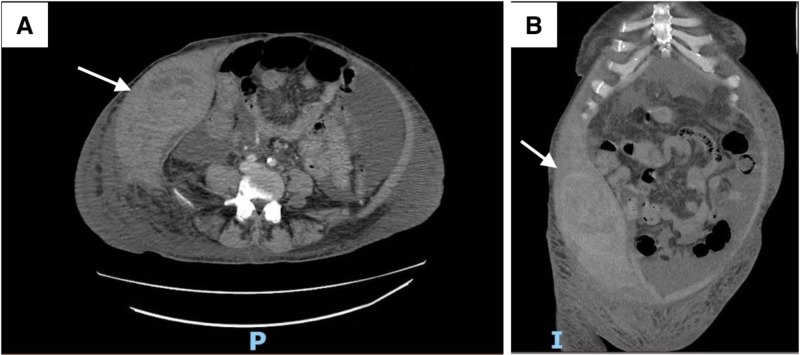
Axial (A) and Coronal view (B) of contrast-enhanced abdominal CT scan showing a large right subcutaneous anterior abdominal wall hematoma measuring 24.4 cm x 94 cm with arterial extravasation.

**Figure 3:**
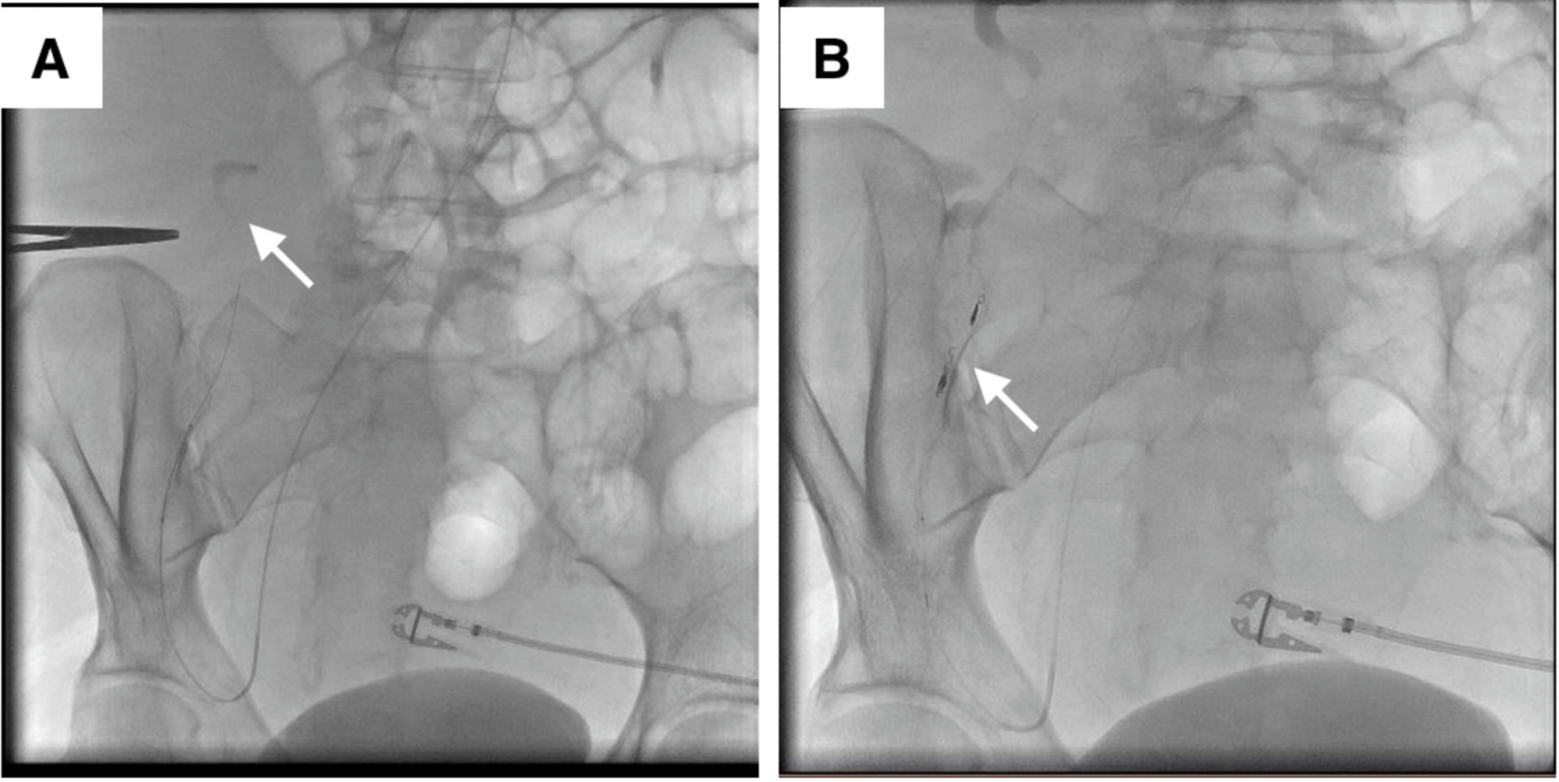
Transcatheter angiography of the right inferior epigastric artery and the ascending branch of the right deep circumflex iliac artery with demonstration of extravasation of contrast medium from the ascending branch of the right deep circumflex iliac artery (A). Resolution of extravasation after transcatheter embolization of the ascending branch of the right deep circumflex iliac artery (B)

The day after diagnostic paracentesis, the patient began reporting sharp pain near the incision site, and her hemoglobin dropped to 6.7 g/dL from a baseline of 8.1 g/dL. She became hypotensive and was transfused two units of packed red blood cells. She underwent an emergent computerized tomography (CT) angiography of the abdomen and pelvis to rule out a retroperitoneal bleed. The CT angiogram demonstrated a large right anterior abdominal wall hematoma measuring 24 cm x 9 cm with evidence of active arterial extravasation primarily from an ascending branch of the right deep circumflex iliac artery and partially from the right inferior epigastric artery (Figure 2). Subsequently, she then underwent emergent interventional radiology (IR) guided transcatheter coil embolization of the bleeding sites via a right-radial approach and therapeutic drainage of the ascitic fluid from the left lower quadrant (Figure 3). 

**Figure 4:**
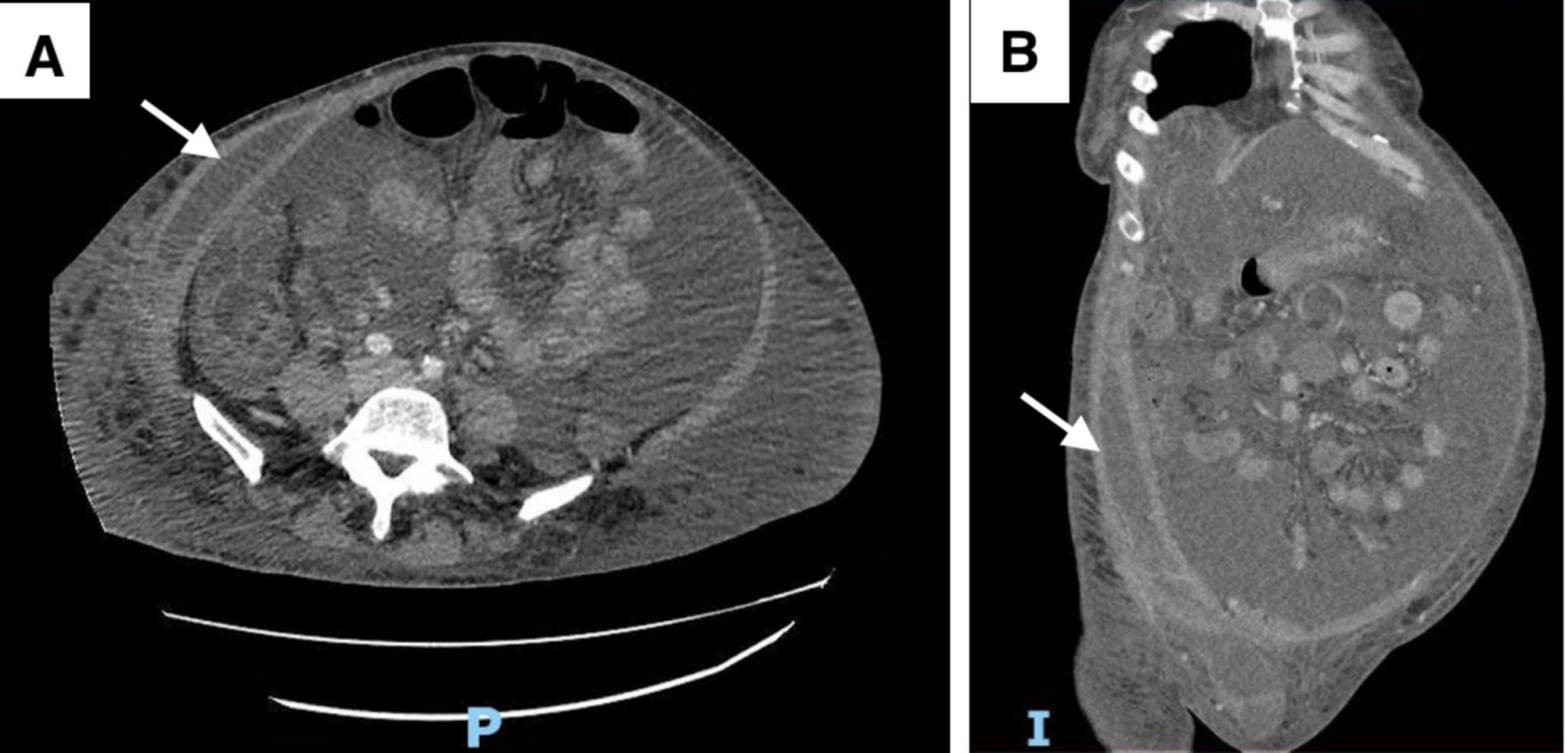
Axial (A) and Coronal view (B) of contrast-enhanced abdominal CT scan showing decreased size and continued evolution of right anterior abdominal wall hematoma status post coil embolization of the right inferior epigastric artery without any evidence of active bleeding.

The patient remained hemodynamically stable without any further drop in hemoglobin. She underwent a repeat CT angiogram of the abdomen two days later, which revealed that the size of the right anterior abdominal wall hematoma had decreased (Figure 4). Ascitic total nucleated cell count was 135/µL (10% granulocytes), and serum ascites albumin gradient was 1.4 g/dL which was consistent with portal hypertension. In the context of low total protein level in the ascitic fluid of 1.2 g/dL, she was started on long term oral ciprofloxacin 500mg per day for spontaneous bacterial peritonitis prophylaxis. The patient remained stable and was discharged three days later. She was accepted as a candidate for liver transplantation.

## Discussion

Aspiration of ascitic fluid via paracentesis is carried out for diagnostic and therapeutic purposes. It can be performed via the landmark-based approach or the more modern ultrasound-guided approach, and the latter option has a more favorable outcome considering the rate of complication was reported to have reduced from 4.7% in the landmark-based approach compared to 1.4% in the sonographic guided method [1]. Sonographic guidance has also proven to reduce hospital length of stay and overall cost [2]. A linear sonographic transducer is preferred in thin patients, and a phased array can be used in the more obese population [2]. The use of a color doppler to determine the location and distribution of the inferior epigastric artery (IEA) and surrounding vessels before accessing the peritoneal cavity is not often routinely performed; however, several studies have documented that this benefit outweighs the risk of injuring the IEA [2,3].

IEA, the proximal branch of the external iliac artery, lies 4-8 cm from the midline abdominal wall, and in 80% of cases, at least one branch of the IEA lies further lateral to the rectus abdominus muscle [4]. IEA punctures the transversalis fascia and traverses anterior to the arcuate line and lies between the rectus abdominus and posterior layer of the rectus sheath with a tortuous course and are highly variable and thereby increasing risk of laceration [5,6]. In patients with a distended abdomen, especially in those with ascites, the distribution of this vasculature can be shifted further laterally. This also applies to the deep circumflex iliac artery (DCIA), the second branch of the external iliac artery, which originates from the lateral aspect of the distal external iliac artery and traverses laterally along the superior border of the iliac crest. A high-frequency transducer can be used by starting at the mid-inguinal ligament and traveling superomedial towards the umbilicus to determine the location of the IEA. In those with ascites, pointing the probe initially at a more lateral location, then moving the probe along the anterior axillary line over the distended abdomen will offer greater success of detecting the IEA and possibly the DCIA [3]. It is now preferred to access the peritoneal cavity via the right and left lower quadrants as they have the least vascularity and thinner walls [7]. Hence, when choosing an appropriate incision site, it is best to opt for a location more than 8 cm from the midline or 3-5 cm medial to the anterior superior iliac spine to avoid piercing the IEA and surrounding vasculature [2,4].

Complication rates for paracentesis are minimal, ranging from 1%-2%, and they include dry tap, bleeding, infection, bowel perforation, and fluid leak [2]. Hemorrhagic complications can be categorized into abdominal wall hematomas, pseudoaneurysms, and hemoperitoneum, and although rare, they carry a high risk of mortality [8]. These most commonly arise from injury to the IEA, and this is a rare case to be reported in the literature with iatrogenic laceration of the DCIA as a result of ultrasound-guided paracentesis. Other causes of injury to the DCIA reported in the literature include those following surgical drain placement, blunt abdominal wall trauma, laparoscopic surgeries, anterior iliac bone graft harvest, and after renal allograft biopsy. An isolated case of pseudoaneurysm of the DCIA due to a complicated tap was also identified in our literature review [9]. 

Laceration of the IEA or surrounding vessels can be managed via bipolar coagulation, tamponade, suturing, open surgery, ultrasound-guided thrombin injection and transcatheter arterial embolization and the latter has been proven to carry a high success rate of 90% across several studies and was also a successful approach for our patient without causing significant mortality [10,11]. 

## Conclusions

Complications from ultrasound-guided paracentesis, although rare, can have devastating consequences to patients undertaking the procedure. This case report sheds light into an entity involving dissection of the DCIA, which is not commonly encountered. Based on our findings, we suggest the more routine use of the color Doppler mode to assess the IEA and surrounding vasculature before cannulation of the peritoneal cavity in order to reduce any risk of morbidity and mortality. Further studies should be carried out to determine the incidence of the aberrant anatomy of abdominal wall vasculature, especially in those with ascites. Additionally, investigations should be carried out to determine if pre-procedure precautions like the color doppler mode could be used to possibly reduce the risk of all paracentesis related morbidity and mortality.
